# Hemostasis in Intracranial Hemorrhage

**DOI:** 10.3389/fneur.2017.00080

**Published:** 2017-03-15

**Authors:** Deepak Gulati, Dharti Dua, Michel T. Torbey

**Affiliations:** ^1^Neurology Department, The Ohio State University College of Medicine, Columbus, OH, USA

**Keywords:** intracerebral hemorrhage, anticoagulants, new oral anticoagulants, reversal of anticoagulation, hemostasis

## Abstract

Spontaneous non-traumatic intracerebral hemorrhage (ICH) is associated with high morbidity and mortality throughout the world with no proven effective treatment. Majority of hematoma expansion occur within 4 h after symptom onset and is associated with early deterioration and poor clinical outcome. There is a vital role of ultra-early hemostatic therapy in ICH to limit hematoma expansion. Patients at risk for hematoma expansion are with underlying hemostatic abnormalities. Treatment strategy should include appropriate intervention based on the history of use of antithrombotic use or an underlying coagulopathy in patients with ICH. For antiplatelet-associated ICH, recommendation is to discontinue antiplatelet agent and transfuse platelets to those who will undergo neurosurgical procedure with moderate quality of evidence. For vitamin K antagonist-associated ICH, administration of 3-factor or 4-factor prothrombin complex concentrates (PCCs) rather than fresh frozen plasma to patients with INR >1.4 is strongly recommended. For patients with novel oral anticoagulant-associated ICH, administering activated charcoal to those who present within 2 h of ingestion is recommended. Idarucizumab, a humanized monoclonal antibody fragment against dabigatran (direct thrombin inhibitor) is approved by FDA for emergency situations. Administer activated PCC (50 U/kg) or 4-factor PCC (50 U/kg) to patients with ICH associated with direct thrombin inhibitors (DTI) if idarucizumab is not available or if the hemorrhage is associated with a DTI other than dabigatran. For factor Xa inhibitor-associated ICH, administration of 4-factor PCC or aPCC is preferred over recombinant FVIIa because of the lower risk of adverse thrombotic events.

Spontaneous non-traumatic intracerebral hemorrhage (ICH) is associated with high morbidity and mortality throughout the world with no proven effective treatment (1). Of the nearly 40,000 Americans who experienced an ICH in 1997, 35–52% were dead within 1 month and only 20% were living independently at 6 months (2). Although the incidence of ICH decreased over the past decade, case fatality and long-term morbidity remained unchanged with reported 30-day ICH mortality ranging from 30 to 50% (3, 4).

## Early Hematoma Growth

Hematoma expansion and early deterioration are common within the first few hours after ICH onset (4). Majority of hematoma expansion occur within 4 h after symptom onset, whereas an additional 12% of patients developed hematoma growth within the next 21 h (5). Early hematoma expansion is reported in 19–38% of patients with ICH and is associated with early deterioration and poor clinical outcome (5–7).

Hematoma growth is seen as a crucial and independent determinant of both mortality and functional outcome after ICH. For each 10% increase in hematoma growth, there is a 5% increased hazard of death, a 16% greater likelihood of worsening by one point on the mRS, or 18% of moving from independence to assisted independence or from assisted independence to poor outcome on the Barthel Index (8). Early hematoma growth has gained a lot of attention as an important cause of early neurological deterioration after ICH and has been thought to be a therapeutic target in many ongoing trials.

## Rationale for Ultra-Early Hemostatic Therapy for ICH

The three most important and consistent predictors of poor outcome after ICH are hematoma volume, presence of intraventricular hemorrhage, and depressed level of consciousness (9–12). Of these, hematoma volume is the single most powerful predictor of 30-day mortality after ICH (12). As bleeding can possibly continue for several hours, it is possible that ultra-early hemostatic therapy may be useful.

In order to even conceive any therapeutic benefit, hemostatic therapy should be given as soon as possible given that hematoma expansion is expected in 10, 17, or 22% of patients after a 15, 30, or 45-min treatment delay following the baseline scan (13). Although the challenges of stroke treatment within a narrow time window are well established, the feasibility of this approach is supported by the fact that ICH patients present to emergency departments earlier than ischemic stroke patients (14).

## Hemostatic Therapeutic Approach

Patients at risk for ICH are with underlying hemostatic abnormalities, which include those taking oral anticoagulant drugs, antiplatelet agents, those with acquired or congenital coagulation factor deficiencies, and those with inherited or acquired qualitative or quantitative platelet abnormalities. Treatment strategy should include appropriate intervention based on the history of use of antithrombotic use or an underlying coagulopathy in patients with ICH.

### Platelet-Related ICH

More than 25% of patients presenting with ICH are taking antiplatelet therapy (15). Observational studies suggest that antiplatelet use before ICH might worsens the outcome by increasing the risk of early hematoma growth (16). Patients taking antiplatelet therapy beforehand have a 27% increased odds of death compared with those not taking antithrombotic drugs (17).

Platelet transfusion is potentially beneficial and used prophylactically in many clinical settings. Observational studies have shown variable association with outcome after platelet transfusion for acute ICH in patients taking antiplatelet therapy (18–20). The effect of platelet transfusion on ICH patients was studied in a recent randomized open label phase 3 trial, Platelet Transfusion versus Standard Care after acute stroke due to spontaneous ICH associated with antiplatelet therapy (PATCH). The study concluded that platelet transfusion was inferior to standard of care for patients taking antiplatelet therapy before ICH (21). The odds of death and dependence at 3 months were higher in the platelet transfusion group (OR 2.05; 95% CI 1.18–3.56) (21). Forty-two percent of participants who received antiplatelet therapy had a serious adverse event during their hospital stay. After the findings of the PATCH trial, platelet transfusion cannot be recommended for the treatment of ICH in patients taking antiplatelet therapy (21). It is important though to keep in mind that most of the participants in PATCH had taken aspirin and relatively few had taken ADP inhibitors (21).

PATCH recent results appear to be contradictory to an earlier randomized trial of platelet transfusion in patients with ICH (22). This prospective, double-blind, parallel, randomized, controlled trial enrolled patients with acute hypertensive basal ganglia hemorrhages undergoing craniotomy and hematoma evacuation. In patients who received platelets, there was less ICH recurrence (14 versus 35%, *P* = 0.02) and lower postoperative hematoma volume in treated groups versus the untreated group. Additionally, those who received platelets had a significant reduction in mortality compared to those who did not (15.5 versus 34.2%, *P* = 0.02). Patients who were aspirin-resistant at baseline and did not receive transfusion had similar outcomes to aspirin-naive patients and better outcomes than aspirin-sensitive patients who did not receive a platelet transfusion. Platelet transfusion carries significant risks with limited benefit in ICH patients (23). Desmopressin (DDAVP) may be an attractive alternative.

DDAVP is an analog of vasopressin. It increases the endothelial release of large factor VIII: von Willebrand factor multimers and may also increase platelet membrane glycoprotein expression, promoting platelet adhesion to the endothelium (24–26). DDAVP has been shown to reduce bleeding time (27) and improved platelet function (as measured by collagen/epinephrine-closure time) (28) following DDAVP administration in uremic patients exposed to aspirin. DDAVP for antiplatelet reversal have been reported in one study (29). Patients with either history of aspirin use or reduced platelet activity received DDAVP 0.4 μg/Kg IV. In this study, DDAVP was well tolerated and improved platelet activity and low rate of hematoma growth after ICH (29). Table 1 summarizes the Neurocritical Care Society recommendation for antiplatelet agent reversal (30).

**Table 1:** **Guidelines for antiplatelet agent reversal**.

(1) Recommend discontinuing antiplatelet agents when in intracerebral hemorrhage (ICH) patients (good practice statement) (2) Suggest against platelet transfusion for patients with antiplatelet-associated ICH who will not undergo a neurosurgical procedure (conditional recommendation, low-quality evidence) (3) Suggest platelet transfusion for patients with aspirin- or ADP inhibitor-associated intracranial hemorrhage who will undergo a neurosurgical procedure (conditional recommendation, moderate quality of evidence) Recommend platelet function testing prior to platelet transfusion if possible (strong recommendation, moderate quality evidence) When platelet function testing is not readily available, empiric platelet transfusion may be reasonable (conditional recommendation, low-quality evidence) Recommend against platelet transfusion for patients with normal platelet function (strong recommendation, moderate quality evidence) (4) Suggest an initial dose of one single-donor apheresis unit of platelets. Platelet testing is suggested prior to repeat platelet transfusion (conditional recommendation, moderate quality evidence) (5) Suggest consideration of a single dose of desmopressin (DDAVP) in ICH (0.4 mcg/kg IV) associated with aspirin/COX-1 inhibitors or ADP receptor inhibitors (conditional recommendation, low-quality evidence)

### Vitamin K-Related ICH

The use of antithrombotic agents has increased over the last decade and is expected to continue to rise over the ensuing decades (31). Vitamin K antagonists (VKAs) are the most common treatments for secondary stroke prevention for patients with atrial fibrillation. Antithrombotic-associated-ICH comprises 12–20% of patients with ICH (32, 33). Intracerebral hemorrhage accounts for the vast majority (90%) of all VKA-related deaths (34). The rate of taking OACs has increased with the aging population and increased use of anticoagulant drugs in recent decades (31, 35). Most ICH related to VKA use occur with international normalizing ratios (INRs) in the recommended therapeutic range, although the risk of hemorrhage is further increased with progressive elevations of the INR (36–38). Urgent reversal of anticoagulation may improve outcomes, reduce mortality, and limit hematoma expansion (39–41).

Several effective treatment options are available for the reversal of VKAs, including vitamin K, fresh frozen plasma (FFP), factor concentrates, and activated factors. FFP, along with vitamin K, has been the mainstay of treatment in U.S. for years, but more recently, prothrombin complex concentrates (PCCs), activated PCC (aPCC) factor VII inhibitor bypassing activity (FEIBA), and recombinant activated factor VIIa (rVIIa) have emerged as potential therapies.

#### Vitamin K

It normalizes the INR by providing the necessary substrate to synthesize necessary clotting factors. Since it takes vitamin K several hours to be effective, it should never be used alone in the emergent treatment of ICH (42). It is important to administer vitamin K as an adjunctive therapy given its long half-life. Higher doses (5–10 mg) of vitamin K reverse INR more quickly than lower doses (43–46). Although this has not been well studied by given the fast rate of hematoma expansion in the first few hours after ICH, a large single dose of vitamin K is preferred to divided dosing (47). Vitamin K is more effective at INR reversal when administered IV as compared to similar dose administered subcutaneously (43, 44). Although oral and IV administration of vitamin K are equally effective in INR reversal, reversal is faster *via* the IV route (43, 45). The infusion should be administered slowly due to the low risk of anaphylaxis (0.03%); however, the benefit of faster infusion and INR reversal justifies the risk (48, 49).

Fresh frozen plasma directly replaces the clotting factors. Although the data regarding its use in ICH are mixed, it is commonly administered with vitamin K (50–54). Given the delay needed to prepare FFP makes its use in acute ICH very limited. Goldstein et al. observed that for every 30 min of delay in the first dose of FFP, the odds of INR reversal within 24 h was decreased by 20% (53). Furthermore, urgent INR reduction was only achieved in 9.6% of patient receiving FFP (55).

Prothrombin complex concentrates are biologically inactivated, vitamin-K-dependent coagulation factors prepared from pooled plasma agents that are lyophilized and can be quickly reconstituted and administered to patients. The first 4-factor PCC (Kcentra, CSL Behring, King of Prussia, PA, USA), containing coagulation factors II, VII, IX, and X, as well as proteins C and S, received FDA approval in 2013 for reversal of coagulopathy from oral VKA therapy in adults with acute major bleeding and those patients requiring urgent surgery or interventional procedures.

The advantages with PCC are no need for cross matching, reconstituted and administered rapidly in a small volume, and does not transmit infectious agents. The disadvantages of FFP are the requirement of thawing and cross matching, allergic and infectious transfusion reactions, large volumes, and unable to achieve rapid correction of INR (53, 56). PCCs rapidly normalize the INR (within minutes) in patients taking warfarin (57–59). The rate of achieving an INR <1.3 within 30 min of completing therapy was 62.2% for PCC and 9.6% for FFP with similar thromboembolic events and fluid overload being more common with FFP (55). PCCs may increase the risk of thrombotic complications, although the risk appears low (57).

There is currently no trial to evaluate 3-factor and 4-factor PCCs against each other. In the international multicenter registry of over 1,500 ICH patients, patients treated with 3-factor PCC demonstrated improved case-fatality compared with those treated with 4-factor PCC (60). The target INR ranges from <1.3 to <1.5 has been cited in various studies (61). A large multinational observational study of VKA-ICH reversal demonstrated no significant differences in the case fatality ratios for patients treated with FFP compared with PCC (60). Several small studies have shown increase survival and reduced hematoma expansion with PCC (39, 41, 62, 63).

The INR Normalization in Coumadin Associated Intracerebral Hemorrhage (INCH) trial, a randomized controlled trial of PCC compared with FFP, was stopped prematurely due to clear benefit of PCC. Results showed that 4-factor-PCC is superior to FFP in normalizing the INR within 3 h in patients with VKA-related ICH (64).

Recombinant FVIIa (rFVIIa) has been shown to correct the INR more rapidly than FFP (65). rFVIIa is not currently recommended for routine use in warfarin reversal. Two large randomized trials evaluated the use of rFVIIa in ICH (66, 67). Although both trials showed that rFVIIa was able to limit hematoma expansion, the phase III trial failed to demonstrate a benefit in functional outcome or mortality with significantly more thrombotic events as compared to placebo (9 versus 4%) (66, 67). Limited data exist comparing PCC to rFVIIa. Current guidelines recommend against the routine use of rFVIIa alone for the reversal of VKAs (30). Table 2 summarizes the NCS recommendations for VKA reversal (30).

**Table 2:** **Guidelines for vitamin K antagonists (VKAs) reversal**.

(1) Discontinue VKAs when ICH is present or suspected (good practice statement) (2) Urgent reversal of VKAs in patients with ICH with the following exceptions (strong recommendation, moderate quality evidence) High suspicion of ICH due to cerebral venous thrombosis (conditional recommendation, very low-quality evidence) In patients with concurrent symptomatic or life-threatening thrombosis, ischemia, heparin-induced thrombocytopenia, or DIC (good practice statement) (3) Administration of vitamin K as soon as possible or concomitantly with other reversal agents (strong recommendation, moderate quality evidence). The following dosing is recommended: One dose of vitamin K 10 mg IV Subsequent treatment should be guided by follow-up international normalizing ratios (INRs) (good practice statement) If repeat INR is still elevated C 1.4 within the first 24–48 h after reversal agent administration, redose with vitamin K 10 mg IV (good practice statement) (4) Administer 3-factor or 4-factor prothrombin complex concentrates (PCCs) rather than fresh frozen plasma (FFP) to patients with INR >1.4 (strong recommendation, moderate quality evidence) 4-factor PCC is preferred over 3-factor PCC (conditional recommendation, low-quality evidence) Suggest initial reversal with PCC alone rather than combined with FFP or recombinant FVIIa (rFVIIa) (conditional recommendation, low-quality evidence) PCC dosing should be weight-based and vary according to admission INR and type of PCC used (strong recommendation, moderate quality evidence) INR testing should be repeated soon after PCC administration (15–60 min), and serially every 6–8 h for the next 24–48 h. Subsequent treatment should be guided by follow-up INR. Repeat PCC dosing may lead to increased thrombotic complications and risk of DIC. If repeat INR is still elevated >1.4 within the first 24–48 h after initial PCC dosing, suggest further correction with FFP. (5) Recommend against administration of rFVIIa for the reversal of VKA (strong recommendation, low quality evidence) (6) If PCCs are not available or contraindicated, alternative treatment is recommended over no treatment (strong recommendation, moderate quality evidence) Treatment with FFP and vitamin K is recommended over no treatment (strong recommendation, moderate quality evidence) Suggest dosing FFP at 10–15 ml/kg IV along with one dose of vitamin K 10 mg IV (conditional recommendation, low-quality evidence)

## New Anticoagulant Medication-Related ICH

New oral anticoagulants (NOACs) including direct thrombin inhibitors (DTIs) and factor Xa inhibitors (FXa-Is) have been recently approved for primary and secondary prophylaxis of thromboembolic conditions. The use of NOAC for systemic anticoagulation has increased dramatically due to their putative advantages versus warfarin, rapidly growing proportion of elderly Americans, and the expanding indications for NOAC medications. NOAC have a shorter half-life (5–15 h), fewer food and drug interactions, fixed dosing, no need for frequent blood monitoring, and a rapid and reliable duration to onset and offset (68).

Direct thrombin inhibitors carry less risk of ICH (69). In the Randomized Evaluation of Long-Term Anticoagulant Therapy (Re-Ly) trial, dabigatran had a 0.31% risk of ICH compared to 0.76% with warfarin (70). NOAC were associated with a 47% odds reduction in the risk of fatal bleeding in one of the meta-analysis of 11 randomized controlled trials compared to vitamin K use (71).

Although animal models have shown some benefit of PCC in reversing the effect of dabigatran (72, 73), a randomized trial of healthy volunteers failed to shown an effect of PCC on aPTT, ecarin clotting time (ECT), and thrombin time (TT) (74). On the basis of *in vitro* and preclinical data, administration of aPCC is preferred over 4-factor PCC for patients receiving dabigatran (75).

Idarucizumab, a humanized monoclonal antibody fragment against dabigatran, completely reversed the anticoagulant activity of dabigatran in 88–98% of patients (76). In a prospective cohort of 90 patients receiving dabigatran who required urgent reversal, 5 g of IV idarucizumab normalized the dilute TT and ECT in 88–98% of patients within minutes. There was one reported thrombotic event within 72 h of administration of the drug. In October 2015, U.S. FDA granted accelerated approval to Praxbind (idarucizumab) for use in patients who are taking the anticoagulant Pradaxa (dabigatran) during emergency situations when there is a need to reverse Pradaxa’s blood-thinning effects.

### Direct FXa-I

The risk of ICH for FXa-I is lower than warfarin (69). In ROCKET-AF (An Efficacy and Safety Study of Rivaroxaban with Warfarin for the Prevention of Stroke and Non-Central Nervous System Systemic Embolism in Patients with Non-Valvular Atrial Fibrillation) trial, rivaroxaban had 0.5% risk of ICH compared to 0.7% with warfarin (77). Similarly, in ARISTOTLE (Apixaban for the Prevention of Stroke in Subjects with Atrial Fibrillation) trial, apixaban had a 0.33% risk of ICH compared with 0.8% with warfarin (78).

Animal studies and limited clinical data suggest that 4-factor PCC is better for patients with refractory rivaroxaban- or apixaban-associated hemorrhage due to high concentrations of factor X contained within (79, 80). One retrospective study reported the use of 4-factor PCC to reverse coagulopathy secondary to rivaroxaban and apixaban in the setting of ICH (80).

Andexanet alfa is a recombinant human protein (modified FXa protein) that binds and inactivates FXa inhibitors. It has been studied with rivaroxaban, apixaban, and low-molecular-weight heparin (81). Infusion of andexanet alfa was able to quickly normalize several laboratory assays in randomized controlled trials of healthy elderly volunteers (82). However, it may require prolonged infusion because anti-FXa activity returned after stopping the infusion. Andexanet has no known hemostatic effects. It did reduce tissue factor pathway inhibitor levels, which may imply a prothrombotic effect (81). The ANNEXA-4 trial demonstrated that an initial bolus and subsequent 2-h infusion of andexanet substantially reduced anti-factor Xa activity in patients with acute major bleeding associated with FXa-I, with effective hemostasis occurring in 79% (83).

Ciraparantag, a synthetic water-soluble compound, binds directly to unfractionated and low-molecular weight heparin. It serves as a potential universal antidote for several different classes of anticoagulant drugs. The synthetic compound ciraparantag may prove to be an effective universal reversal agent. It was able to reduce bleeding in animal models and normalize coagulation assays in healthy volunteers given edoxaban and dabigatran with a single IV dose (81, 84). Results of further studies and review by the FDA are pending.

If oral drug intake was within a couple hours of presentation, oral activated charcoal offers a low side effect treatment option for NOAC (85). However, caution should be exhibited given the risk of aspiration and potential elevated intracranial pressure induced by vomiting. Hemodialysis should also be considered. Hemodialysis is more effective with dabigatran and less so for rivaroxaban or apixaban because they are more highly protein bound (85). Table 3 summarizes the NCS guidelines for reversal of NOAC (30).

**Table 3:** **Guidelines for reversal of new oral anticoagulants**.

**Direct factor Xa inhibitors (FXa-Is)**
(1) Discontinue FXa-I when ICH is present or suspected (good practice statement) (2) Pharmacological reversal of oral FXa-I should be guided primarily by bleeding (major or intracranial) and not primarily by laboratory testing (conditional recommendation, low-quality evidence) (3) Suggest administration of activated charcoal (50 g) to intubated ICH patients with enteral access and/or those at low risk of aspiration who present within 2 h of ingestion of an oral direct factor Xa inhibitor (conditional recommendation, very low-quality evidence) (4) Suggest administering a 4-factor prothrombin complex concentrate (PCC) (50 U/kg) or activated PCC (aPCC) (50 U/kg) if ICH occurred within 3–5 terminal half-lives of drug exposure or in the context of liver failure (conditional recommendation, low-quality evidence) (5) Suggest administering 4-factor PCC or aPCC over recombinant FVIIa (rFVIIa) because of the lower risk of adverse thrombotic events (conditional recommendation, low-quality evidence)
**Direct thrombin inhibitors (DTIs)**
(1) Discontinue DTI when ICH is present or suspected (good practice statement) (2) Pharmacological reversal of DTI should be guided primarily by bleeding (major or intracranial) and not primarily by laboratory testing (conditional recommendation, low-quality evidence) (3) Suggest administering activated charcoal (50 g) to intubated intracranial hemorrhage patients with enteral access and/or those at low risk of aspiration who present within 2 h of ingestion of an oral direct thrombin inhibitor (conditional recommendation, very low-quality evidence) (4) Recommend administering idarucizumab (5 g IV in two divided doses) to patients with ICH associated with dabigatran if dabigatran was administered: Within a period of 3–5 half-lives and there is no evidence of renal failure (strong recommendation, moderate quality of evidence) or There is renal insufficiency leading to continued drug exposure beyond the normal 3–5 half-lives (strong recommendation, moderate quality of evidence) (5) Suggest administering aPCC (50 U/kg) or 4-factor PCC (50 U/kg) to patients with ICH associated with DTI if idarucizumab is not available or if the hemorrhage is associated with a DTI other than dabigatran (6) In patients with dabigatran-associated ICH and renal insufficiency or dabigatran overdose, suggest hemodialysis if idarucizumab is not available (conditional recommendation, low-quality data) (7) In patients with dabigatran-associated ICH who have already been treated with idarucizumab, PCC, or aPCC, with ongoing evidence of clinically significant bleeding, we suggest consideration of redosing idarucizumab and/or hemodialysis (Conditional recommendation, low-quality evidence) (8) Recommend against administration of rFVIIa or FFP in direct thrombin inhibitor-related intracranial hemorrhage (strong recommendation, low-quality evidence)

## Conclusion

Intracerebral hemorrhage is a devastating disease associated with high morbidity and mortality. Although no clinical trials have clearly showed the benefit of prompt reversal of coagulation abnormalities, it is a good practice to correct these abnormalities as fast as possible until further data are available.

## Author Contributions

DG and DD worked on the literature search and prepared the initial draft of the manuscript. MT reviewed the literature and edited the manuscript significantly. DG and MT finalized the manuscript.

## Conflict of Interest Statement

The authors declare that the research was conducted in the absence of any commercial or financial relationships that could be construed as a potential conflict of interest.
